# Correlations of Aerobic Capacity with External and Internal Load of Young Football Players during Small-Sided Games

**DOI:** 10.3390/s24072258

**Published:** 2024-04-01

**Authors:** Yiannis Michailidis

**Affiliations:** Laboratory of Evaluation of Human Biological Performance, Department of Physical Education and Sports Science, Aristotle University of Thessaloniki, New Buildings of Laboratories, University Campus of Thermi, 57001 Thessaloniki, Greece; ioannimd@phed.auth.gr; Tel.: +30-23-1099-2248

**Keywords:** correlation, soccer, running performance, oxygen consumption, small-sided games

## Abstract

Aerobic capacity plays a crucial role in football performance, making it a focal point in training processes. Small-sided games (SSGs) are widely used in football training, but the relationship between aerobic capacity and running performance during SSGs remains unclear. The aim of this study was to investigate possible correlations between maximum oxygen uptake (VO_2_max) and running performance in youth football players in SSGs (4:4, 3:3, 2:2, 1:1) with three different pitch sizes per player (150, 100, 75 m^2^/player). Sixteen male U15 football players participated in the study. Players underwent the Yo-Yo intermittent recovery test level 1, and their VO_2_max was estimated based on their performance. Subsequently, players participated in SSGs wearing GPS devices to measure internal and external load. Pearson or Spearman correlation was applied for statistical analysis depending on the normal distribution of the data. The results reveal that, for 4:4 and 3:3 relationships, larger pitches led to a greater impact of aerobic capacity (total distance (TD): 4:4, 150 m^2^/pl, r = 0.715, *p* = 0.002; 100 m^2^/pl, r = 0.656, *p* = 0.006; 75 m^2^/pl, r = 0.586, *p* = 0.017). In the 2:2 relationship, the opposite was observed, with more correlations appearing on smaller pitches (TD: 2:2, 100 m^2^/pl, r = 0.581, *p* = 0.018; 75 m^2^/pl, r = 0.747, *p* < 0.001). In the 1:1 relationship, correlations with VO_2_max, total distance, and speed were observed only on the larger pitch. In conclusion, the aerobic capacity of young football players can influence running performance indicators in SSGs. Therefore, aerobic capacity could serve as a criterion for team composition, making SSGs more competitive. Additionally, the variation in correlations in the 2:2 relationship and their limited presence in the 1:1 relationship may be attributed to technical–tactical factors, such as increased ball contacts and one-on-one situations typically occurring in smaller setups.

## 1. Introduction

Football is a particularly popular sport engaged in by millions of athletes [1]. The majority of these athletes are children participating in football academies. A recent study reports that the number of boys in the developmental age engaged in football in Europe surpasses three million [2]. Football is popular in childhood due to its simple rules and the minimal skills required to play (at a basic level).

Small-sided games (SSGs) are widely used in football academies. These are football games with a small number of players in smaller playing areas. Some of the key advantages of SSGs include simulating actual matches, simultaneously training technical–tactical elements with physical elements, thereby saving valuable time for coaches [3,4]. Additionally, they are versatile and do not require special equipment to implement. Finally, they are enjoyable for participants as they are games and do not exhibit the constraints of exercise.

However, SSGs also present some disadvantages. Specifically, it is more challenging to determine load characteristics such as covered distance and intensity [5] compared to running exercises. Changes in field dimensions, the number of players, and rules can influence the internal and external load on players, affecting coaching outcomes [5,6].

The issue of load control has been addressed in recent years through the use of technology. Specifically, the development of Global Positioning Systems (GPSs) allows coaching staff to monitor both the real-time internal load (e.g., heart rate) and external load (e.g., total distance—running performance) of each football player. The use of these systems has decrypted the physical demands of a football match [7]. This has led sports scientists to seek performance factors (e.g., aerobic capacity—VO_2_max) that could enhance the running performance of football players.

From the literature, it appears that some studies have explored the relationship between performance in aerobic tests or VO_2_max and the running performance of football players in matches. In one of the initial studies on this topic [8], researchers observed correlations between the performance of professional football players in field tests (Yo-Yo intermittent recovery test, multi-stage fitness test) and the high-intensity running distance covered by the players in their matches. Similar findings were reported in a later study [9]. However, in another study wherein VO_2_max was measured, no correlation with running performance in the match was observed [10]. In a study conducted on developmental-age football players, a correlation was observed between performance in the Yo-Yo intermittent recovery test and running performance in the match, albeit with low explanatory power (R^2^ = 17–22%) [11].

However, all the above studies have investigated the potential relationship between performance in physical fitness tests and running performance in matches. What happens during training with SSGs? Can aerobic performance indicators influence external and internal load during SSGs? What is the impact of these indicators in relation to the size of the playing area and the number of players? These questions have led to the idea of the present study.

Therefore, the aim of this study is to investigate the relationship between VO_2_max estimated in young football players using the Yo-Yo intermittent recovery test level 1 and the external and internal load in SSGs in three different playing areas with four different player ratios (1:1, 2:2, 3:3, 4:4). The results of the study will assist coaches in directing their training. Specifically, it will reveal which playing area size influences aerobic capacity in each match ratio (1:1, 2:2, 3:3, 4:4).

## 2. Methods

### 2.1. Experimental Design

This study was conducted during the competition period. All measurements were taken on a plastic grass field and were performed at least 48 h after the athletes’ previous training or match. Initially, anthropometric measurements (height, weight, percentage of body fat) were taken (Seca 220e, Seca, Hamburg, Germany; Lafayette Instrument, Lafayette, IN, USA), followed by a maximum sprint test for 40 m (Witty, Microgate, Bolzano, Italy). The results of this test were used to adjust the GPS zones (10 Hz Polar Team Pro, Kempele, Finland). Subsequently, the Yo-Yo intermittent recovery test level 1 was conducted to estimate maximum oxygen uptake (VO_2_max). The two tests above (40 m sprint and Yo-Yo intermittent recovery test level 1) were conducted only at the beginning of the study. Over the next three weeks, the football players trained with small-sided games (SSGs) in 4:4, 3:3, 2:2, and 1:1 ratios. Different pitch areas (150 m^2^/player, 100 m^2^/player, and 75 m^2^/player) were used in the SSGs. During each training session, players wore a GPS sensor (the same in each session), preceded by a 15 min warm-up. After the warm-up, teams were formed, and SSGs were conducted. After a 5 min recovery, and at the end of the training, GPS data were collected and compiled in an Excel file.

### 2.2. Participants

Sixteen male, young football players from an amateur academy participated in the study (age (years) = 15.0 ± 0.4; Height (cm) = 172.0 ± 4.9; weight (kg) = 65.6 ± 8.6; body fat (%) = 20.4 ± 3.5; VO_2_max (ml·kg^−1^·min^−1^) = 45.9 ± 3.1). They trained three times a week and also participated in a match. The inclusion criteria were as follows: (a) participation in ≥90% of the training sessions, (b) no musculoskeletal injuries in the last 6 months, (c) participation in all SSGs. Participants and their guardians were briefed on the study’s goals, benefits, and potential risks. They then signed a consent form. The study received approval from the local Institutional Review Board (approval number 190/2024) in accordance with the principles outlined in the Helsinki Declaration.

### 2.3. Anthropometric Measurements

Body weight was measured with a precision of 0.1 kg, and height with a precision of 0.1 cm, using a stadiometer and a Seca 220e scale (Seca 220e, Seca, Hamburg, Germany). During measurements, players wore only their underwear. Body fat percentage was measured using the skinfold method with a Lafayette skinfold caliper (Lafayette Instrument, Lafayette, IN, USA). More specifically, using the Lafayette skinfold caliper (Lafayette Instrument, Lafayette, IN, USA), four skinfolds (biceps, triceps, suprailiac, subscapular) were measured on the right side of the football players’ bodies. The obtained values were then utilized in the Siri equation [12] for the estimation of body fat percentage.

### 2.4. Speed Measurements

For the measurement of the players’ speed, a maximum speed test of 40 m was conducted. Specifically, the football players ran as fast as they could from a standing position over a distance of 40 m. The time it took for them to cover this distance was recorded by two pairs of photocells (Witty, Microgate, Bolzano, Italy) placed at the start (0 m) and at 40 m. The players started 0.3 m behind the starting line, while the photocells were positioned at a height of 0.6 m to avoid measurements from being influenced by arm movement. Each football player made two attempts with a 3 min recovery, and for statistical analysis, the best performance was considered. The coefficient of variation for this test ranged around 3.6%. The results of this speed test were used to determine above which speed movement the sprint would be defined (the fifth zone). From the results, it was evident that this speed for the entire group of children in the study was 19 km/h.

### 2.5. Yo-Yo Intermittent Recovery Test Level 1 (YYIR1) and VO_2_max

The YYIR1 is an intermittent intensity-increasing test wherein the football player runs according to an auditory signal. Specifically, when the player hears the sound signal, they start from the starting line and run 20 m. Upon hearing the second sound signal, they must have crossed the line (at 20 m) and turned (180°) toward the starting line. At the third sound signal, they should have passed the starting line. Then, they have 10 s to run/walk 5 m backward and return to the starting line for the next sound signal. As the test progresses, the running speed increases. In case a player cannot follow the auditory signals for two consecutive times, the test ends for that player, and their performance is based on the last completed run. The following equation proposed by Bangsbo et al. (2008) [13] was used to estimate VO_2_max:

VO_2_max (mL/kg/min) = YYIR1 distance (m) × 0.0084 + 36.4

### 2.6. External Load

The external load in all SSGs was measured using the Global Positioning System (GPS) from Polar (10 Hz Polar Team Pro, Kempele, Finland). The speed zones (z1–5) utilized were z1: 0.10–6.99 km/h; z2: 7.00–10.99 km/h; z3: 11.00–14.99 km/h; z4: 15.00–18.99 km/h; z5: >19.00 km/h. For accelerations and decelerations, only “intense” ones were considered, specifically accelerations > 2 m/s² and decelerations < −2 m/s². External load indices used for correlations included total distance (TD), pace (m/s), distance at different speed zones (Dz1-5), number of sprints (Sn), and the number of accelerations (Accn) and decelerations (Decn). As mentioned earlier, to avoid measurement errors due to internal variability among GPS sensors, each football player had a specific transmitter throughout the study. The GPS sensors were placed in the special pocket on the t-shirt provided by the Polar company, between the player’s shoulder blades.

### 2.7. Heart Rate

The heart rate (HR) of the soccer players during SSGs was recorded in real time using the Polar Team Pro 5.41(Kempele, Finland).

### 2.8. Small-Sided Games (SSGs)

**Figure 1:**
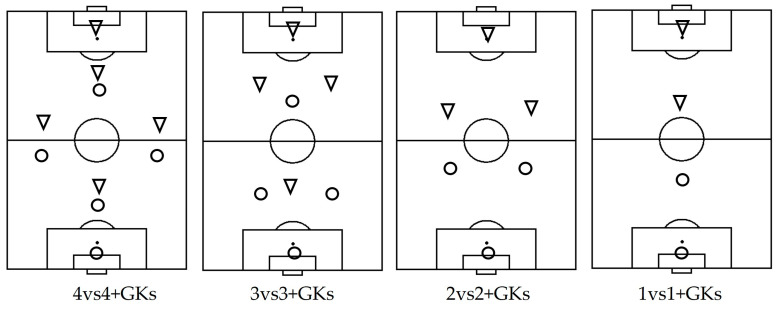
Positioning of football players during SSGs. Symbols: Circle—the attacking team; Triangle—the defending team.

As mentioned earlier, the formations used in the SSGs were 4:4, 3:3, 2:2, and 1:1 with goalkeepers. In each training session, the team members for the SSGs were determined randomly. More specifically, a lottery with random numbers was used to determine the teams (for all relationships). Additionally, the composition of each team was recorded to ensure that the teams were different each time. The initial positioning of the football players at the start of the SSG is shown in Figure 1. In all formations, four repetitions were applied. The duration of each repetition in 4:4 was 4 min, in 3:3 was 3 min, in 2:2 was 2 min, and in 1:1 was 1 min. The recovery interval between repetitions in all ratios was 3 min, except for the 1:1 ratio where the interval was 2 min. Each of the ratios (4:4, 3:3, 2:2, 1:1) used player-to-space ratios of 150 m²/player, 100 m²/player, and 75 m²/player. The field dimensions are presented in Table 1.

### 2.9. Statistical Analysis

Initially, descriptive statistics were conducted, and the results are presented as mean values ± standard deviation. Normal distribution was assessed using the Shapiro–Wilk test. For data following normal distribution, the Pearson correlation test was applied, while for non-normally distributed data, the Spearman’s correlation test was used. According to Hopkins [14], the magnitude of the correlation coefficient was considered trivial (r < 0.1), small (0.1 < r < 0.3), moderate (0.3 < r < 0.5), large (0.5 < r < 0.7), very large (0.7 < r < 0.9), and nearly perfect (r = 1.0). Significance was defined at *p* < 0.05.

## 3. Results

**Figure 2:**
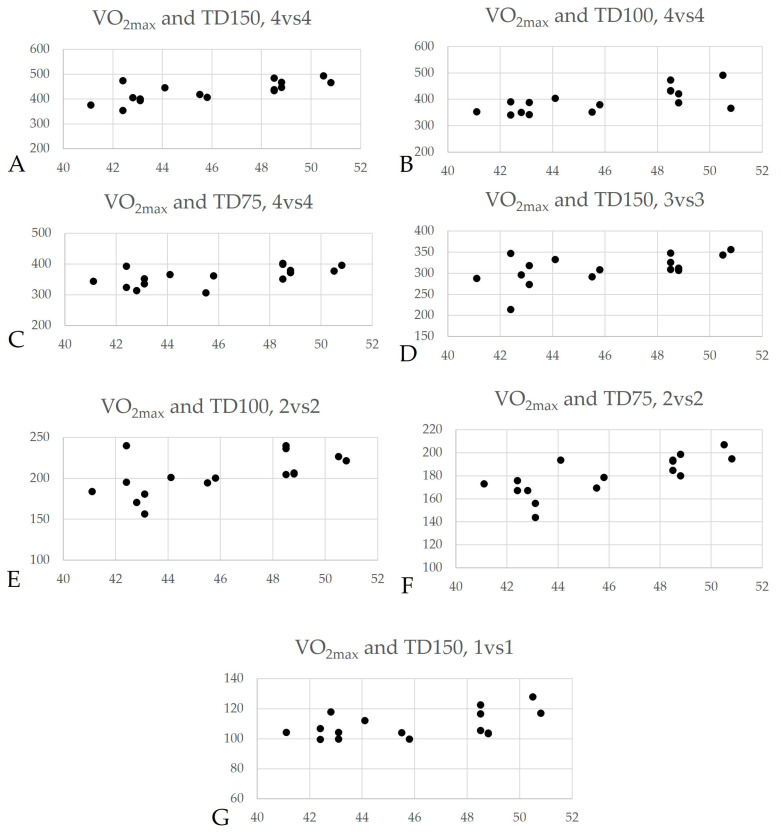
Scatter plots showing correlations between VO_2_max and total distance (TD). (**A**) Scatter plot for VO_2_max with total distance in relation to 4vs4 (150 m^2^/player). (**B**) Scatter plot for VO_2_max with total distance in relation to 4vs4 (100 m^2^/player). (**C**) Scatter plot for VO_2_max with total distance in relation to 4vs4 (75 m^2^/player). (**D**) Scatter plot for VO_2_max with total distance in relation to 3vs3 (150 m^2^/player). (**E**) Scatter plot for VO_2_max with total distance in relation to 2vs2 (100 m^2^/player). (**F**) Scatter plot for VO_2_max with total distance in relation to 2vs2 (75 m^2^/player). (**G**) Scatter plot for VO_2_max with total distance in relation to 1vs1 (150 m^2^/player).

From the results, it was evident that, in the 4:4 ratio, VO_2_max correlated in all three dimensions with the parameters of TD, pace, and distances in zones z3 and z4. Specifically, for TD, a very large correlation was observed (r = 0.715, *p* = 0.002) in the ratio of 150 m^2^/player, decreasing as the playing area diminished (r = 0.656, *p* = 0.006 for 100 m^2^/player and r = 0.586, *p* = 0.017 for 75 m^2^/player). Scatter plots showing correlations between VO_2_max and total distance (TD) are presented in Figure 2. A similar trend was observed in the pace parameter (r = 0.714, *p* = 0.002 for 150 m^2^/player, r = 0.653, *p* = 0.006 for 100 m^2^/player, and r = 0.592, *p* = 0.016 for 75 m^2^/player). Concerning the distances covered in zones z3 and z4, large and very large correlations with VO_2_max were observed in all three dimensions of the studied fields.

In the 3:3 ratio, VO_2_max showed large correlations with several parameters on the playing field at a ratio of 150 m^2^/player; meanwhile, as the space decreased, the number of correlated parameters decreased. Thus, while in the ratio of 150 m^2^/player, seven correlations were observed; in the ratio of 100 m^2^/player, four correlations were observed; and in the ratio of 75 m^2^/player, only two were found, where VO_2_max positively correlated with the distance in z4 (r = 0.620, *p* = 0.010) and negatively with the average heart rate (HRavg) (r = −0.504, *p* = 0.046).

In the 2:2 ratio, the number of VO_2_max correlations showed the opposite pattern to the 3:3 ratio. In the large ratio of 150 m^2^/player, only two correlations were observed (HRavg r = −0.504, *p* = 0.046 and z4 r = 0.602, *p* = 0.010); in the ratio of 100 m^2^/player, four correlations were observed; and in the ratio of 75 m^2^/player, nine correlations were found. The only parameter that was found to correlate with VO_2_max in all three dimensions was the distance in z4 (r = 0.602, *p* = 0.014 for 100 m^2^/player and r = 0.667, *p* = 0.005 for 75 m^2^/player).

In the 1:1 ratio, correlations were observed only on the playing field with the ratio of 150 m^2^/player. VO_2_max correlated with TD (r = 0.624, *p* = 0.017), pace (r = 0.624, *p* = 0.017), and the distance covered in z2 (r = 0.579, *p* = 0.030). Detailed correlations for all variables are presented in Table 2. In the Supplementary Files, a table is provided displaying the mean values and confidence intervals of the variables utilized in the study.

## 4. Discussion

The purpose of this study was to investigate whether there are correlations between VO_2_max, estimated using the YYIR1 test, with performance parameters in small-sided games (SSGs) of four different ratios and three different field sizes. The main findings were as follows: (1) In the 4:4 ratio, VO_2_max correlated with performance parameters in all three dimensions of the fields. (2) In the 3:3 ratio, VO_2_max influenced several performance parameters on the large field (150 m^2^/player), but as the playing area decreased, these correlations diminished. (3) In the 2:2 ratio, VO_2_max correlated with several parameters on the small field, but as the field size increased, these correlations disappeared. (4) In the 1:1 ratio, correlations appeared only on the large field.

As mentioned in the introduction, there are no studies that have explored the relationship between VO_2_max and performance indicators in SSGs. Therefore, reference will be made to studies that have investigated the relationship with running performance during matches.

SSGs are games used by coaches at all levels and constitute a fundamental part of their training units. The main advantage they present is that players are trained simultaneously in technical–tactical aspects while improving physical fitness elements [15]. Regarding physical fitness, the SSGs (ranging from 4:4 to 1:1) studied in this research are used for different purposes. Specifically, larger ratios are used for developing specific aerobic capacity, while smaller ratios are usually employed for power development in football players [16].

Starting with the 4:4 ratio, it was observed that more correlations appeared on the large field, and the number of correlations decreased as the m^2^/player ratio decreased. Significant and very significant correlations of VO_2_max with GPS parameters were observed in all three dimensions, specifically with parameters such as TD, pace, and distances covered in zones z3 and z4. Notably, for the TD and pace indices in the 150 m^2^/player ratio, a very high correlation was observed, with the correlation coefficient decreasing as the m^2^/player ratio decreased.

From these findings, it seems that the space where SSGs are applied can influence parameters related to the physical condition of football players. Additionally, players with better aerobic capacity cover more distance at a higher pace. This conclusion is supported by GPS data, showing that the total distance covered on the large field was 432 m, on the medium field it was 395 m, and on the small field it was 361 m. The movement pace was 106, 98, and 90 m/s, respectively. Greater distances in larger fields are consistent with the existing literature [17,18]. The higher intensity on the larger field is also confirmed by the average heart rate, which was 89% on the large field and 84% on the other two dimensions. This higher intensity on larger fields is consistent with previous studies [18,19].

Furthermore, in the 150 and 100 m^2^/player ratios, a significant negative correlation was observed between the distance covered in zone z1 (walking) and VO_2_max. On the large field, correlations with the number of Acc and Dec were also observed. The increased number of intense Acc and Dec on the large field is consistent with earlier studies [19,20,21]. Therefore, young football players with better aerobic capacity walk less during SSGs, benefiting more in the area of physical fitness and experiencing greater neuromuscular fatigue from Acc and Dec.

In the 3:3 ratio, the observed correlations had a similar pattern to the 4:4 ratio. That is, most correlations appeared on the large field, and as the space decreased, the number of correlations also decreased. A notable observation is that very large correlations were not observed in this relationship. Additionally, there were no parameters that showed a correlation with VO_2_max in all three field sizes. On the large field, it was observed that higher VO_2_max was associated with more distance running, and in zones z2 and z3 they also ran at a higher speed, performed more sprints, and more decelerations.

In the 100 m^2^/player ratio, VO_2_max showed correlations with the distance covered in high-speed zones (z4, z5) and with the number of sprints and decelerations. Finally, in the 75 m^2^/player ratio, two significant correlations were observed: one positive with the distance covered in zone z4 and one negative with the average heart rate. From these findings, it could be inferred that, as the space decreases, fewer parameters are affected by aerobic capacity. The GPS data indicated that the SSG on the large field had the highest intensity (88% HRmax), with the other two fields showing similar intensity (around 83% HRmax). Similar to the 4:4 ratio, the higher intensity on larger fields is confirmed by previous studies [19,20]. Also, on the large field, the greatest distance was covered (311 m) with the highest pace (104 m/s). Previous studies reported greater distances on larger fields [22].

In the 2:2 condition, most correlations of VO_2_max were observed in the small field while, as the size of the fields increased, the number of correlations decreased. Therefore, in this condition, the opposite pattern was observed compared to the two larger conditions (4:4 and 3:3). In the small and medium fields, correlations were observed with TD, pace, and distances covered in zones z3 and z4. In the small field, a positive correlation was also observed with the number of sprints, accelerations (Acc), and decelerations (Dec). In the large field, a positive correlation was observed with the distance covered in zone z4 and a negative correlation with the average heart rate. Thus, while in smaller fields, aerobic capacity seems to correlate with several running performance parameters, and in the large field, correlations may be limited, possibly because aerobic mechanisms are more critical. The larger space favors intense attacking actions (sprints in open space), which mainly rely on the anaerobic mechanism of energy production. Looking at the GPS data on the small field, it is noted that the average intensity (87% of HRmax) is higher than that on the large field (84% of HRmax). This finding contrasts with previous research where higher intensities were observed on larger fields [23].

In the 1:1 condition, the only correlations observed were on the large field and involved TD, pace, and the distance covered in zone z2. As mentioned earlier, the 1:1 ratio in football is used to improve players’ power, meaning that actions during these SSGs activate the anaerobic mechanism. In this condition, higher values for both TD and pace were observed on the large field. These findings are in line with previous studies [24].

Examining the impact of the number of players at the same m^2^/player ratio (e.g., 150 m^2^/player), differences were observed in the correlation of VO_2_max with running performance indicators. These differences are pronounced when transitioning from the bigger fields 4:4 and 3:3 to smaller fields 2:2 and 1:1. The variation in the players’ tactics to cope with all four phases of the game (attacking, defensive transition, defending, offensive transition) seems to affect their running profile [25]. Another factor that can influence the correlation results is the exercise time, which varied in each condition (4:4, 3:3, 2:2, 1:1), although the exercise time-to-player ratio remains constant and equal to 1. In the 100 m^2^/player ratio, the most correlations of VO_2_max with GPS indicators were observed.

As previously mentioned, the Yo-Yo IR1 test proposed by Bangsbo et al. (2008) [13] was used to measure aerobic capacity. However, it should be noted that, in their study, the authors do not provide information about the characteristics of the sample (*n* = 141) they used (e.g., gender, age), which are factors that could influence the results. Therefore, this particular equation may not be suitable for measuring VO_2max_ in the participants of the present study, and this fact should be taken into consideration when evaluating the results. Additionally, by using the equation, estimation rather than measurement of VO_2max_ is conducted, indicating that the VO_2_max value estimated using the equation may differ from the actual value. For this reason, it is also cited as one of the limitations of the study.

As previously mentioned, there are no studies exploring the correlation of physical fitness indicators with running performance of football players during SSGs; therefore, references are made to studies related to matches. Castagna et al. (2010) [8] observed significant correlations between YYIR1 test performance and high-intensity efforts during the match, similar to findings from other researchers [26]. However, there are studies that did not find significant correlations between physical fitness indicators and running performance [11]. Differences observed in research may be attributed to age differences in the sample and variations in the speed zones used [11].

This study has some limitations. Firstly, VO_2_max was not directly measured but estimated using a field test. Power analysis was not conducted for the estimation of sample size, and it is considered that the sample was small, which, particularly in correlation studies, may impact the results and their generalization [14]. Also, in this study, the same exercise durations were not used for each relationship. This choice was made to make the exercise durations realistic (as used in training for 1:1, 2:2), but it limits the ability to make comparisons between relationships (1:1 vs. 2:2). Finally, no recording of tactical movements during SSGs was conducted, which may allow for the identification of the impact of players’ tactics on their running performance. Future research addressing these limitations will provide additional information to coaches regarding the use of SSGs and the relationship between running performance and fitness indicators.

## 5. Conclusions

In conclusion, regarding the field sizes in the 4:4 and 3:3 conditions, it was observed that, the larger the field, the more running performance depends on aerobic capacity. In the 2:2 condition, the greatest impact of aerobic capacity was observed on smaller fields, while in the 1:1 condition, correlation was observed only on the large field. Smaller ratios involve more ball contacts and one-on-one duels, elements that can influence the running profile. Additionally, the running profile may be influenced by tactical variations in conditions with fewer players (e.g., limited overlap).

The significant positive correlations indicate that higher aerobic capacity is associated with covering longer distances and a more intense pace. From a coaching perspective, this knowledge can assist coaches in grouping their players based on their aerobic capacity, making teams more competitive during SSGs, and allowing players to derive greater benefits in terms of physical fitness. Moreover, in the 3:3 and 4:4 conditions, large fields impose greater demands on aerobic capacity. It is also noteworthy that, in the 1:1 condition, primarily used for developing players’ strength, fewer correlations with aerobic capacity were observed.

## Figures and Tables

**Table 1:** Space characteristics of small-sided games.

SSG	75 m^2^/Player	100 m^2^/Player	150 m^2^/Player
1:1 + GK	10 × 15 m	20 × 10 m	20 ×15 m
2:2 + GK	20 × 15 m	27 × 15 m	30 × 20 m
3:3 + GK	25 × 18 m	30 × 20 m	36 × 25 m
4:4 + GK	27 × 22 m	32 × 25 m	40 × 30 m

SSGs, small-sided games; GK, goal keeper.

**Table 2:** Correlation indices.

		4:4			3:3			2:2			1:1
		150 m^2^/pl	100 m^2^/pl	75 m^2^/pl	150 m^2^/pl	100 m^2^/pl	75 m^2^/pl	150 m^2^/pl	100 m^2^/pl	75 m^2^/pl	150 m^2^/pl
TD	r	0.715	0.656	0.586	0.532	–	–	–	0.581	0.747	0.624
	*p*	0.002	0.006	0.017	0.034				0.018	<0.001	0.017
Pace	r	0.714	0.653	0.592	0.535	–	–	–	0.582	0.749	0.624
	*p*	0.002	0.006	0.016	0.033				0.018	<0.001	0.017
z1	r	−0.622	−0.528	–	−0.523	–	–	–	–	−0.500	–
	*p*	0.010	0.036		0.038					0.048	
z2	r	–	–	–	0.512	–	–	–	–	0.506	0.579
	*p*				0.043					0.046	0.030
z3	r	0.717	0.760	0.542	0.503	–	–	–	0.508	0.758	–
	*p*	0.002	<0.001	0.030	0.047				0.045	<0.001	
z4	r	0.662	0.643	0.723	–	0.656	0.602	0.620	0.602	0.667	–
	*p*	0.005	0.007	0.002		0.006	0.010	0.010	0.014	0.005	
z5	r	–	0.557	–	–	0.567	–	–	–	–	–
	*p*		0.025 Sp			0.022					
Acc	r	0.658	–	–	–	–	–	–	–	–	–
	*p*	0.006									
Dec	r	0.547	–	–	0.521	0.602	–	–	–	–	–
	*p*	0.028			0.038	0.014					
Sprint	r	–	–	–	0.544	0.497	–	–	–	0.548	–
	*p*				0.029	0.050				0.028 Sp	
HRav	r	–	–	–	–	–	−0.504	−0.504	–	–	–
	*p*						0.046	0.046			

TD, total distance; Pace, m/s; z1 distance covered in zone 1 (0.10–6.99 km/h); z2 distance covered in zone 2 (7.00–10.99 km/h); z3 distance covered in zone 3 (11.00–14.99 km/h); z4 distance covered in zone 4 (15.00–18.99 km/h); z5 distance covered in zone 5 (>19.00 km/h); Acc, accelerations; Dec, decelerations; HRav, heart rate average; Sprint, Spearman test; – denotes no significant correlations.

## Data Availability

Data are available upon request from the corresponding author. Also, files are uploaded in the Supplementary Files.

## Supplementary Materials

The following supporting information can be downloaded at: https://www.mdpi.com/article/10.3390/s24072258/s1, Table S1: Mean values and confidence intervals of the variables utilized in the study.
